# Congenital cytomegalovirus screening in neonates born after 35 weeks gestation—is targeted screening beneficial?

**DOI:** 10.3389/fped.2025.1510612

**Published:** 2025-02-13

**Authors:** Mary Liza Aldon, Gayatiri Raveentheran, Zubair Amin, Si Min Chan, Rie Aoyama, Nancy Tee, Sau Yoke Ng, Chun Kiat Lee, Pick Gate Ng, Jia Ming Low

**Affiliations:** ^1^ Department of Neonatology, Khoo Teck Puat-National University Children Medical Institute, National University Healthcare System, Singapore, Singapore; ^2^ Department of Paediatrics, Yong Loo Lin School of Medicine, National University of Singapore, Singapore, Singapore; ^3^ Department of Laboratory Medicine, National University Health System, Singapore, Singapore

**Keywords:** congenital deafness, sensorineural hearing loss, neonates, small for gestational age, newborn screening

## Abstract

**Introduction:**

Congenital Cytomegalovirus (cCMV) is the most common congenital viral infection and leading cause of non-genetic sensorineural hearing loss in children. Timely treatment within the first months of life can prevent hearing loss. We evaluated utility, feasibility, and short-term cost implications of targeted cCMV screening in a tertiary public healthcare institution in Singapore.

**Methods:**

We analyzed data from neonates born at ≥35 weeks gestation from 1st February 2022 to 31st January 2023, at the National University Hospital, Singapore. Screening criteria for CMV testing were neonates who failed hearing screening at birth, had a birth weight <2.5 kg, small for gestational age (SGA), or had clinical suspicion for cCMV. Urine samples were obtained within two weeks of birth for CMV polymerase chain reaction (PCR) testing. We collected clinical, ophthalmological, neuroimaging, laboratory, and audiological data. A limited, short-term cost analysis was performed to compare costs of three cCMV screening strategies (universal, targeted, and no screening) two years after implementation.

**Results:**

Out of 5,277 neonates, 305 (6%) met the screening criteria. Among those screened, 70.5% (215/305) were SGA or had a birth weight <2.5 kg, and 23.3% (71/305) failed the hearing screen. The overall diagnostic yield was 0.06% (3/5,277). There was one false positive result (0.3%; 1/305). Three neonates (0.98%; 3/305) were diagnosed with cCMV by urine PCR. One was SGA without intracranial abnormality, and two had low birth weight with intracranial calcifications. None had cytopenia or transaminitis, and all ophthalmology screenings were normal. All infected neonates were started on Valganciclovir. One neonate failed the hearing test at one month of age but passed repeat assessments by three months. Two neonates exhibited upper motor neuron signs in the lower limbs and are on long-term follow-up. The total estimated direct treatment-related cost without cCMV screening was S$104,445.79. In the targeted screening model, the total cost was S$146,656.30, compared to S$853,890.16 in the universal screening model.

**Conclusion:**

We demonstrated the feasibility of targeted cCMV screening, which complements universal newborn hearing screening in a tertiary neonatal center in Singapore. Targeted cCMV screening can be implemented cost-effectively during the neonatal period, enabling early detection and intervention.

## Introduction

Congenital cytomegalovirus (cCMV) is the most common congenital viral infection (1) with an overall birth prevalence between 0.6%–6.1%. (2–4) At birth, although most infants are asymptomatic (5), 10%–15% of these infants develop long-term sequelae (1, 3, 6). cCMV is the leading cause of non-genetic sensorineural hearing loss (SNHL) in children (2, 3, 7–9). It accounts for 21% of infants with hearing loss at birth (10), and 25% of deafness by 4 years (2, 9). cCMV also results in neurodevelopmental delay ranging from psychomotor and cognitive disabilities (3, 7) to visual impairment (11), causing a significant economic burden (5, 12).

cCMV is a common infection in Singapore with more than 80% maternal IgG seroprevalence (13, 14). In the past, limited awareness by both healthcare providers and parents, the lack of effective antiviral therapy, and the absence of a rapid and inexpensive diagnostic test contributed to the absence of cCMV screening program in Singapore (15). There is a renewed interest to introduce cCMV screening in Singapore, driven by the success of antiviral treatment in symptomatic infants and the recognition that early intervention with antiviral therapy during the critical language and speech acquisition period improves outcomes (13, 16–18). Encouragingly, a recent local study reported favourable parental attitudes towards universal cCMV screening (19).

We implemented targeted cCMV screening for all neonates born after 35 weeks gestation at our center. We integrated this screening into the universal newborn hearing screening program to detect congenital hearing loss. This approach enabled us to assess the effectiveness of a targeted strategy in identifying neonates with cCMV-related hearing loss, as well as those showing early signs of cCMV at birth. Our aim is to evaluate the utility, feasibility, and short-term cost implications of targeted cCMV screening in a tertiary public healthcare institution in Singapore.

## Methods

This was a prospective study. We analyzed data from neonates born at ≥35 weeks’ gestation from 1st February 2022 to 31st January 2023 at the National University Hospital, Singapore. Secondary outcomes of interest were maternal risk factors associated with cCMV infection; type, duration and potential adverse effects of antiviral therapy; neonatal morbidities including SNHL, neurological, visual impairments; and the rate of follow-up.

**Figure 1:**
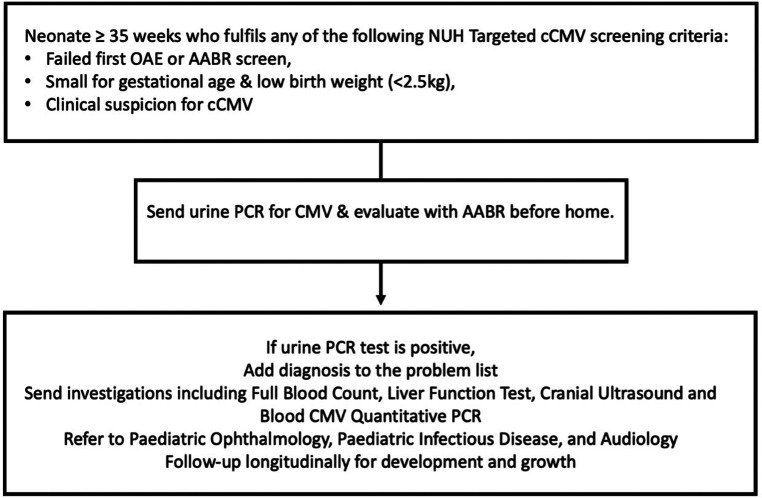
Targeted newborn screening protocol for cCMV at the national university hospital of Singapore. CMV, cytomegalovirus; PCR, polymerase chain reaction; AABR, automated auditory brainstem response.

All neonates with gestational age ≥35 weeks who failed hearing screen at birth, were small for gestational age (SGA) or with a birth weight less than 2.5 kg, or with clinical suspicion for cCMV infection were included (Figure 1). We defined symptomatic cCMV according to the European Society for Paediatric Infectious Diseases guidelines 2017 (17), which included physical examination findings of SGA, microcephaly or neurologic signs. We excluded neonates with gestational age <35 weeks at birth or who were screened for cCMV after three weeks of age. Institutional ethics board approved the study (Domain Specific Review Board reference: 2022/00752).

We collected urine via sterile cotton balls placed in a urine bag within two weeks from birth for CMV polymerase chain reaction (PCR) testing (8, 20, 21). Samples were processed using CMV ELITe MBG Kit on ELITe InGenius System with a detection limit of 220 copies/ml. We defined a positive result for cCMV infection as more than 220 copies/ml in the urine. Notably, blood CMV testing has a limit of detection 90 IU/ml and borderline result is defined as CMV DNA detected but <180 IU/ml. Neonates with positive urine CMV results were closely reviewed to determine if they developed clinically apparent disease.

Clinical decisions on further evaluation and treatment of cCMV-infected infants were made by managing physicians with inputs from pediatric infectious disease specialists. As per our practice cCMV-infected neonates undergo baseline evaluation including full blood count, liver function test and a cranial ultrasound and are evaluated for cCMV-related ophthalmological complications. Symptomatic cCMV-positive neonates are treated with 6-months of Valganciclovir (17), with 2–4 weekly blood test monitoring and a repeat cranial ultrasound. They are also closely followed up outpatient for ophthalmological and auditory sequelae. Hearing assessment is performed with otoacoustic emissions and automated auditory brainstem response. We use Alarcon Score, a validated prognostic tool for infants with cCMV (22). This scoring system includes neuroimaging components and results of hearing screenings and correlates the score with severity of morbidity or mortality.

We extracted the following data from electronic medical records: maternal and neonatal demographics, gestational age, birth weight, length, head circumference, indications for cCMV screening and test results, symptoms, hearing screen results, blood tests and imaging, and treatment duration and complications. We analyzed the data using descriptive statistics to calculate proportions. We summarized the data as count [percentage] and mean [standard deviation (SD)] or median [interquartile range (IQR)] depending on the normality of distribution.

We performed limited, short-term cost analysis to compare cost differences of three strategies of cCMV screening (i.e., universal, targeted and no screening). These included costs involved in screening and treating all cCMV. Major cCMV-related morbidities and the costs associated with further treatment and follow-up were also incorporated into the analysis, up until two-years-old (23). Screening cost of CMV PCR processing of urine sample was S$150.00/sample, excluding nursing manpower and ancillary cost of collecting urine. The cCMV diagnostic evaluation was assumed to be 100% accurate. Infants diagnosed with cCMV were assumed to undergo a medical evaluation and receive treatment as per our unit's cCMV protocol (Figure 1). We calculated the cost required for a comprehensive medical evaluation which includes a panel of blood tests and auditory and ophthalmological evaluation. The analysis included costs of follow-up with the cost of oral Valganciclovir therapy and monitoring for drug toxicity of all cCMV-positive infants (Tables 1, 2) (24). Follow-up costs include cost of consultations, cranial ultrasound, two-to-four weekly blood tests monitoring, a one-time eye review and 6-monthly auditory screenings until two-years-old (25). For unidentified cCMV infants who develop hearing loss, the costs of undiagnosed hearing loss requiring cochlear implants were calculated. The costs associated with a proportion of these infants at risk of neurodevelopmental delays and requiring early intervention with physiotherapy were included. All cost estimations, both in-patient and out-patient, were based on unsubsidised rate and expressed in Singapore dollars (1 USD = 1.34 SGD).

**Table 1:** Detailed breakdown of cost analysis for different strategies of cCMV screening.

Screening strategy	Cost categories	Number of neonates affected	Breakdown of costs	Sum
No screening	Cost of screening	0	150	0
	Cost of treating	0	492	0
	Cost of undiagnosed hearing loss	2	49,590	99,180
	Cost of physiotherapy	2.1	2,507.52	5,265.79
	Total sum of no screening			**104,445.79**
Targeted screening	Cost of screening	305	150	45,750
	Cost of investigating cCMV positive infants—blood tests, cranial ultrasound, eye review	3	492	1,476
	Cost of treatment with 6 months of oral Valganciclovir	3	1,845.12	5,535.36
	Cost of follow-up and monitoring for toxicity	3	1,692.80	5,078.4
	Cost of undiagnosed hearing loss	1.7	49,590	84,303
	Cost of physiotherapy	1.8	2,507.52	4,513.54
	Total sum of targeted screening			**146,656.30**
Universal screening	Cost of screening	5,277	150	791,550
	Cost of investigating cCMV positive infants—blood tests, cranial ultrasound, eye review	26	492	12,792
	Cost of treatment with 6 months of oral Valganciclovir	3	1,845.12	5,535.36
	Cost of follow-up and monitoring for toxicity	26	1,692.8	44,012.8
	Cost of hearing loss and physiotherapy for undiagnosed	0	0	0
	Total sum of universal screening			**853,890.16**

Total number of neonates from February 2022—January 2023, 5277. Neonates identified via targeted screening criteria—305. cCMV prevalence in general newborn population and targeted study population of high-risk infants were assumed to be 0.5% and 0.98% respectively.

The bold values represent the total cost for each individual screening strategy.

**Table 2:** Components of cost analysis for cCMV screening, evaluation and treatment.

Screening	Urine CMV test	150
Evaluation	Blood investigations—full blood count, liver function test, Serum CMV PCR	268
	Ultrasound cranium	157
	Eye screening	67
	Total	492
Treatment	Valganciclovir 60 mg/ml suspension $9.61 per ml	1,845.12
	For 2 kg baby, assuming 6 months of oral Valganciclovir 60 mg/ml with dose of 16 mg/kg/dose (BD dosing), conservatively total 6 months would cost at least $1,845.12. Price will increase as baby gains weight and dose increases.	
Outpatient costs – until 2 years old
	Follow-up consult costs—first repeat cranial ultrasound, and blood test monitoring 2–4 weekly for first 6 months of life	1,232
	Eye screening follow-up—first review	47
	Audio consult with hearing tests (otoacoustic emission) done 6-monthly until 2 years old and tympanogram once	[60 + 38.6] × 4 + 19.40 = 413.8
	Total	1,692.80
Complications	Cochlear implant device, bilateral	49,000
	Audio consult with hearing tests (otoacoustic emission) and tympanogram, 6 monthly until cochlear implantation (2.9 years old)	[60 + 38.6 + 19.40] × 5 = 590
	Total	49,590
	Physiotherapy for neurodevelopmental delays—monthly until 2 years of age, assuming outpatient 45min-1 h duration	104.48 × 24
	Total	2,507.52

Based on an estimated cCMV prevalence (2, 4), 0.5% of the neonates were assumed to be cCMV-positive (26/5,277). Of these, we assumed an estimated 10% of cCMV-positive infants to be symptomatic and therefore require treatment (3, 4, 17). 7.5% of cCMV-positive infants were assumed to have significant hearing loss requiring cochlear implants, yielding the final number 2 out of 26 infants. This estimation was based on a previously published study suggesting 15% of cCMV infants develop SNHL, of which 50% develop severe bilateral hearing loss (26). According to another study, the mean age for cochlear implantation in cCMV-positive infants is 2.9 years and we calculated follow-up costs up to two-years-old (27). We estimated 8% of cCMV-positive infants would develop motor or speech delay and require therapy (28). We assumed universal screening was capable of identifying all cCMV-positive cases.

## Results

### 3.1 Baseline characteristics of screened neonates and their mothers

**Figure 2:**
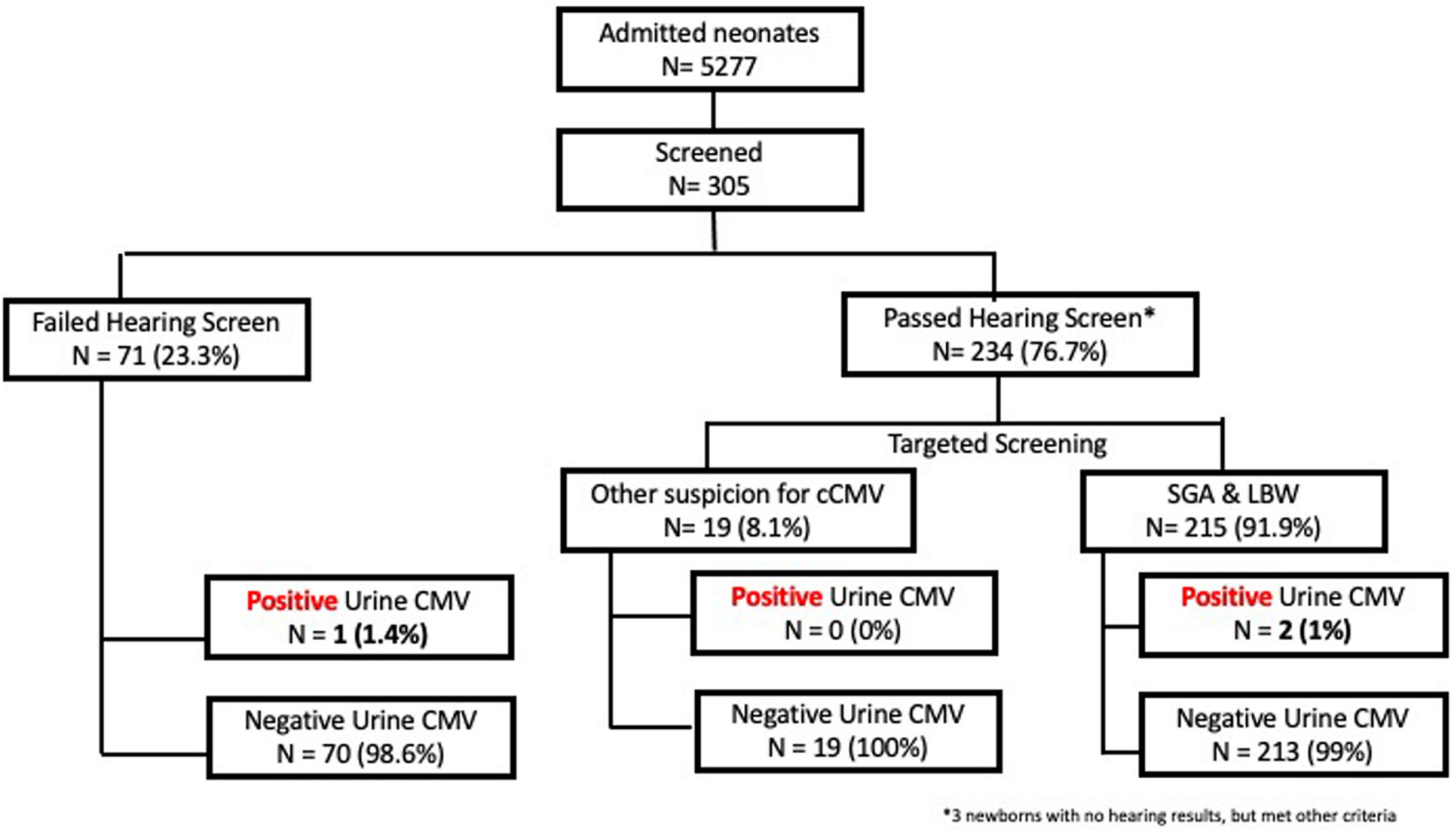
Comparing universal hearing-based versus targeted cCMV screening.

From 1st February 2022 to 31st January 2023, 5,277 neonates were born at the National University Hospital, Singapore. Three hundred and five neonates met the inclusion criteria for cCMV screening (Figure 2). The mean gestational age of the neonates screened was 38.28 (IQR 37.4, 39.1) weeks with a mean birthweight of 2,571 (±474.5) grams. Majority of neonates were Chinese and Malay (39.3%, *n* = 120/305 and 40.3%, *n* = 123/305, respectively). The median maternal age was 31 (IQR 28, 34) years. 46.6% (142/305) of mothers were multiparous. 10.2% (31/305) of mothers underwent serological testing for CMV, out of which 38.7% (12/31) were CMV IgG positive (Table 3).

**Table 3:** Demographic characteristics of mothers and neonates screened for cCMV at national university hospital from 1st of February 2022 to 31st January 2023 (*n* = 305).

Characteristics	*N* (%)
Male	129 (42.3)
Female	176 (57.7)
Maternal age in years (median, interquartile range)	31 (28, 34)
Multiparous	142 (46.6)
Maternal CMV status	
Unknown	274
Negative (IgG and IgM negative)	19
Primary Infection (IgM positive)	0
CMV Immune (IgG positive with high avidity)	12
Race	
Chinese	120 (39.3)
Malay	123 (40.3)
Indian	35 (11.5)
Others	27 (8.9)
Mean gestational age (median, interquartile range)	38.28 (37.4, 39.1)
Gestational age	
Preterm (<37 weeks)	40 (13.1)
Term (≥37 weeks)	265 (86.9)
Birth weight centile	
<10th centile	194 (63.6)
10th-90th centile	108 (35.4)
>90th centile	3 (1)
Occipital-frontal head circumference Centile	
<10th centile	129 (42.5)
10th–90th centile	168 (54.9)
>90th centile	7 (2.2)
No data (out-born)	1 (0.3)
Neonatal hearing screen result (AABR/OAE)	
Unilateral Failed	20 (6.6)
Bilateral Failed	51 (16.7)
Passed	231 (75.7)
No data (out-born)	3 (1)
SGA symmetrical	104 (34.1)
SGA asymmetrical	90 (29.5)
Low birth weight <2.5 kg	21 (6.9)
Other screening indications	
Microcephaly	29 (9.5)
Macrocephaly	2 (0.7)
Intracranial abnormalities	14 (4.6)
Others e.g., intrauterine growth restriction, generalized skin erosions, perinatal depression concerns of intrauterine infection	3 (1.0)
> One indication for cCMV screening	30 (9.8)

### 3.2 Indications for screening

All 305 neonates who met the inclusion criteria were screened following the targeted screening protocol (Figure 2). Among these neonates, 23.3% (71/305) failed a hearing screen. Of the remaining neonates, 91.9% (215/234) were SGA or had a low birth weight of less than 2.5 kg, and 8.1% (19/234) had other clinical suspicion of cCMV (e.g., microcephaly, abnormal antenatal head scan, or intrauterine growth retardation). 70.5% (215/305) neonates were either SGA or had a birth weight <2.5 kg, regardless of hearing test status. Among these neonates, 48.4% (104/215) were symmetrical SGA. 9.8% (30/313) neonates had more than one indication for CMV screening (Table 3).

### 3.3 Baseline characteristics of neonates with cCMV, treatment course and their subsequent follow-up

Among those screened, 0.98% (3/305) were diagnosed with cCMV. The overall diagnostic yield for the entire population was 0.06% (3/5,277). We excluded one infant with false positive result who had an inconclusive initial test result but a confirmatory second sample sent within the first week of life was negative.

The median gestational age of the infected neonates was 37 (IQR 37, 37.2) weeks, mean birthweight was 2,333 (±135.8) grams, and mean head circumference was 33 (±1) cm (Table 4). Two out of three were born to mothers with frequent occupational exposures to young children (22–24). The third neonate was born in another hospital and transferred to our institution for phototherapy with limited information on maternal age, occupation, and hearing test results at birth. Only one out of three neonates had maternal CMV serological testing during pregnancy.

**Table 4:** Neonates with positive CMV test results (*n* = 3, excluding one false positive).

Characteristics of CMV positive newborns	Baby 1	Baby 2	Baby 3	Average
Race	Chinese	Chinese	Malay	
Gestational age (weeks + days) (median, interquartile range)	37 + 0	37 + 0	37 + 3	37 (37, 37.2)
Birth weight (gm, percentile)	2,350 (13%)	2,460 (13%)	2,190 (4%)	2,333 (±135.8)
Occipital frontal circumference (cm, percentile)	33 (52%)	34 (68%)	32 (20%)	33 (±1)
Maternal age (years)	–^a^	33	33	33
Maternal occupation	–^a^	General practitioner	Pre-school teacher	–
Newborn hearing screen	–^a^	Passed	Failed both OAE & AABR	–
Blood CMV	Negative at 1 month of age	Positive at 2 weeks of age (<180 IU/ml viral load); negative by 1 month of age	Negative at 4 months of age	–
Neuroimaging CrUS MRI	CrUS: left basal ganglia calcification CrUS (repeat): Left basal ganglia calcification appear smaller and less distinct	CRUS: scattered tiny echogenic foci in bilateral cerebral hemispheres. MRI: few small foci of susceptibility seen in the supratentorial reflecting calcification/hemorrhagic foci	CrUS normal	–
Alarcon score	1	1	0	
Ophthalmological evaluation	Normal	Normal	Normal	
Liver function test	Normal	Normal	Normal	
Absolute Neutrophil Count (10^9^/L)	2.14	0.52	2.56	2.59 (±0.32)
Treatment with Valganciclovir				
Initiation of Valganciclovir treatment	3 weeks of life	2 weeks of life	3 weeks of life	2.6 (±0.6) weeks of life
Duration of treatment	24 weeks	11 weeks	3 weeks	25.6 (±4.0) weeks
		-stopped in between	-stopped due to costs	
Follow-up for adverse effects				
Hearing	Normal	Failed hearing at 1 month, passed by 3 months	Normal	–
Neurology	Normal	Upper motor neuron signs in lower limbs	Upper motor neuron signs in lower limbs	–

Descriptive statistics were performed with data summarized as count (percentage) and mean [standard deviation (SD)] for categorical and continuous variables, respectively. CrUS, Cranial ultrasound. MRI, Magnetic Resonance Imaging.

^a^ Data not available as out born.

Of the three infected neonates, two had central nervous system involvement and required treatment. The remaining neonate was treated despite having mild disease after shared decision-making with parents (Table 4). All three infants were on the mild-unaffected spectrum based on the Alarcon score. One neonate had normal neuroimaging while two had intracranial calcifications detected on surveillance cranial ultrasounds or magnetic resonance imaging of the brain. None of the neonates had cytopenia or liver transaminitis at baseline. One of the infected neonates had viraemia with less than 180 IU/ml viral load in blood CMV testing.

All three infected neonates were started on oral Valganciclovir at 16 mg/kg twice daily dosing. The plan was for six months of treatment with regular follow-up for clinical monitoring, compliance and side effects (17). The first neonate completed the entire treatment course. The other two neonates did not complete the prescribed duration of treatment. One completed 11 weeks of treatment; Valganciclovir was stopped when she developed probable drug-associated neutropenia, with the lowest absolute neutrophil counts of 0.52 × 10^9^/L. The other neonate had three weeks of treatment; after which it was stopped due to logistical and cost constraints.

One-year outcomes of cCMV-positive infants revealed that one neonate had failed initial hearing screen at birth, and one had failed hearing screen at one month of age, but all had normal repeat hearing assessment from three months of age (Table 4). All had normal initial and subsequent follow-up ophthalmological evaluations. Two of the three infants initially showed neurological abnormalities, diagnosed by paediatric neurologists, in the form of upper motor neuron signs that could not be explained by other factors. Neurological abnormalities normalized after physiotherapy (Table 4).

### 3.4 An initial cost analysis comparing no screening, targeted screening and universal screening strategies

**Figure 3:**
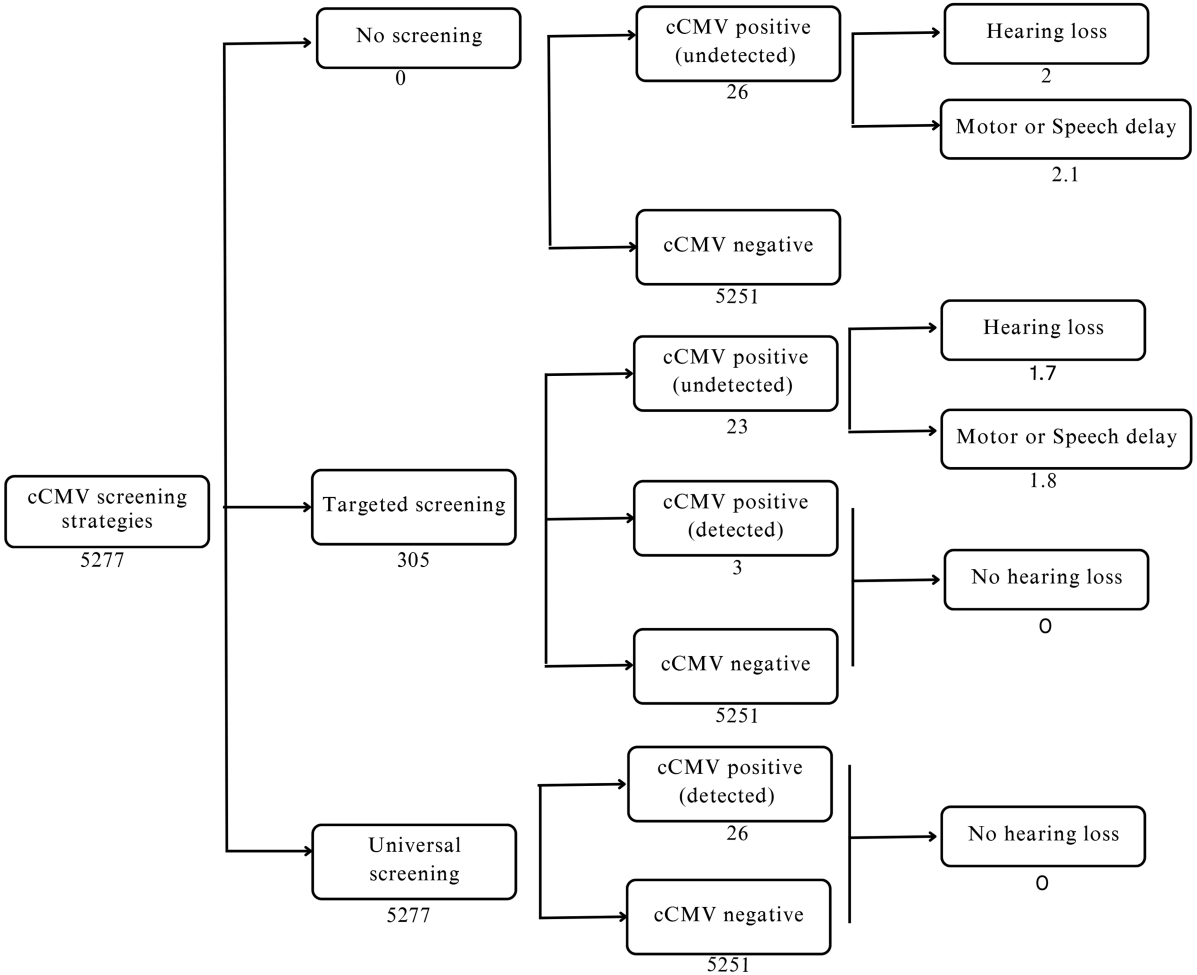
Cost analysis for different strategies of cCMV screening.

A limited short-term cost analysis was performed to compare cost differences amongst universal, targeted and no cCMV screening strategies (Figure 3). Without any screening, we estimated that the total direct cost to be S$104,445.79, primarily from untreated cCMV-positive infants who subsequently develop severe hearing loss requiring cochlear implants, and from those who experience motor or speech delay necessitating therapy. The estimated total cost for universal screening model was S$853,890.16, with costs derived mainly from cCMV-positive neonates undergoing further evaluation, treatment with Valganciclovir and follow-ups and monitoring for toxicity. The estimated total cost for the targeted screening model was S$146,656.30 (Table 1).

## Discussion

In our study, all the three positive cCMV neonates were SGA or had low birth weight, which is similar to a study done by Lorenzoni et al. (29). As compared to other studies on targeted screening for cCMV which mainly screened neonates based on failed newborn hearing screening, our study included other risk factors (e.g., SGA and other clinical suspicions for cCMV) in screening neonates.

The gold standard for cCMV diagnosis is via PCR (30, 31). Viral cultures are associated with high costs, require special storage and transport facilities and are not adaptable for large-scale screening (5). Testing saliva specimens are confounded by false positives from cervico-vaginal secretions and breastfeeding (32). There is also ongoing interest to incorporate cCMV screening with dried blood spot tests (33), however studies reported low sensitivity of this method (34, 35). Urine collection is an attractive option as it is non-invasive, simple to collect and sensitive (8, 20, 21, 36). As such, we chose urine CMV PCR screening within the first two weeks of life to differentiate between congenital vs. postnatal CMV infection as recommended by Ross et al. (21)

cCMV screening meets many of the screening criteria including it being an important health problem; there is a recognizable latent or early preclinical stage; tests are available, simple, validated and acceptable; and treatment/intervention is available and improves outcome (5, 37, 38). There is evidence of potential benefit from antiviral therapy for children with hearing loss at birth (5). There is also evidence that earlier detection together with nonpharmacological interventions improves outcomes for children with delayed hearing loss occurring between 9 and 24 months of age (16, 39).

Our study further strengthens the view that targeted screening is feasible and can be integrated with existing newborn hearing screening—a finding similar to other previous studies (37, 38, 40). cCMV is a common cause of sensorineural hearing loss and early identification allows for earlier intervention and treatment. This integration can be done with little to no additional administrative costs (23).

Several studies recommend targeted screening models similar to our study (41, 42). Targeted screening model focuses on neonates who failed the hearing screen at birth, were small for gestational age (SGA), had a birth weight less than 2.5 kg, or had clinical suspicion of cCMV infection (43, 44). Some studies also suggest including preterm infants due to higher cCMV prevalence among them (29, 45).

Implementing cCMV screening can improve the quality of life for cCMV-positive infants and reduce the economic burden of the disease (12, 46). Studies have described high lifetime costs associated with cCMV-related disabilities, including need for assistive devices, medical costs, special education, and lost productivity (47, 48). At birth, most infants are asymptomatic, but 10%–15% develop long-term sequelae. Universal screening would help identify additional asymptomatic cCMV-positive infants not included in targeted screening and prevent severe complications with timely interventions (49). Oral antiviral therapy is effective, albeit costly in some countries and needs regular follow-up for compliance and complications (16).

A long-term cost savings analysis (23), including averted costs associated with long-term disabilities like hearing loss, would further support universal screening. Although there are concerns that universal screening may cause anxiety in families, studies have shown that parents generally accept screening programs (50). Overall, any screening program is beneficial. The success of a good screening program is tied to adequate access to proper intervention at an affordable level. We believe universal screening is plausibly the most pragmatic as a starting point in countries where cCMV screening is not routine.

Based on our preliminary cost analysis, no cCMV screening might have appeared to be the most cost-saving strategy from a pure short-term economic standpoint. However, it is important to note the minor cost differences between the targeted and no screening strategies, especially when compared to the cost of universal screening. We must also consider the cost savings achieved by preventing cCMV-related disabilities such as hearing or neurological impairments, which in turn improves the quality of life (51). Another benefit that needs to be factored in is avoiding a diagnostic dilemma for cCMV infected children who are born with nonspecific symptoms or who are asymptomatic at birth and subsequently develop disabilities (5). Avoiding such diagnostic dilemma could reduce parental anxiety and stress.

In our study, two out of three neonates did not complete the recommended duration of treatment, one citing cost and financial limitations and another from side-effects from Valganciclovir treatment. Hence even with increasing evidence of benefit of cCMV treatment, there should be detailed counselling of parents on the expected course, benefits and side effects of treatment, and treatment costs.

We would like to highlight several shortcomings of this report. Firstly, our analysis is currently limited to major morbidities associated with cCMV. In calculating the cost, we made few assumptions like the rate of cCMV if universal screening were adopted, the rate of uptake of antiviral treatment, and success rate and complications following such therapy. We assumed cCMV treatment to be 100% effective with no complications. We did not perform long-term cost-benefit analysis. We also excluded quality of life and disability adjusted life years from this estimation (18). Another limitation is that the cost analysis did not account for parental, family, societal burden of having a child with cCMV-related disabilities such as hearing loss or neurodevelopmental disability. Lastly, although maternal CMV serology taken during pregnancy was reported, the exact trimester at which testing was done is unknown.

## Conclusion

Our study showed that targeted cCMV screening is feasible and it complements universal newborn hearing screening in our unit. In the absence of a universal cCMV screening program, targeted cCMV screening should be implemented in the neonatal period to allow for early detection and intervention.

.

## Data Availability

The original contributions presented in the study are included in the article/Supplementary Material, further inquiries can be directed to the corresponding author.

## References

[1] Manicklal S Emery VC Lazzarotto T Boppana SB Gupta RK. The “silent” global burden of congenital cytomegalovirus. Clin Microbiol Rev. (2013) 26(1):86–102. 10.1128/CMR.00062-12 23297260 PMC3553672

[2] Marsico C Kimberlin DW. Congenital cytomegalovirus infection: advances and challenges in diagnosis, prevention and treatment. Ital J Pediatr. (2017) 43(1):38. 10.1186/s13052-017-0358-8 28416012 PMC5393008

[3] Dollard SC Grosse SD Ross DS. New estimates of the prevalence of neurological and sensory sequelae and mortality associated with congenital cytomegalovirus infection. Rev Med Virol. (2007) 17(5):355–63. 10.1002/rmv.544 17542052

[4] Kenneson A Cannon MJ. Review and meta-analysis of the epidemiology of congenital cytomegalovirus (CMV) infection. Rev Med Virol. (2007) 17(4):253–76. 10.1002/rmv.535 17579921

[5] Cannon MJ Griffiths PD Aston V Rawlinson WD. Universal newborn screening for congenital CMV infection: what is the evidence of potential benefit? Rev Med Virol. (2014) 24(5):291–307. 10.1002/rmv.1790 24760655 PMC4494732

[6] Kettler M Shoup A Moats S Steuerwald W Jones S Stiell SC American academy of audiology position statement on early identification of cytomegalovirus in newborns. J Am Acad Audiol. (2023) 34(03/04):084–9. 10.1055/s-0043-1768036 36972678

[7] Boppana SB Ross SA Fowler KB. Congenital cytomegalovirus infection: clinical outcome. Clin Infect Dis. (2013) 57(suppl 4):S178–81. 10.1093/cid/cit629 24257422 PMC4471438

[8] Chiopris G Veronese P Cusenza F Procaccianti M Perrone S Daccò V Congenital cytomegalovirus infection: update on diagnosis and treatment. Microorganisms. (2020) 8(10):1516. 10.3390/microorganisms8101516 33019752 PMC7599523

[9] Morton CC Nance WE. Newborn hearing screening — a silent revolution. N Engl J Med. (2006) 354(20):2151–64. 10.1056/NEJMra050700 16707752

[10] Barbi M Binda S Caroppo S Ambrosetti U Corbetta C Sergi P. A wider role for congenital cytomegalovirus infection in sensorineural hearing loss. Pediatr Infect Dis J. (2003) 22(1):39. 10.1097/00006454-200301000-00012 12544407

[11] Coats DK Demmler GJ Paysse EA Du LT Libby C. Ophthalmologic findings in children with congenital cytomegalovirus infection. J Am Assoc Pediatr Ophthalmol Strabismus. (2000) 4(2):110–6. 10.1067/mpa.2000.103870 10773810

[12] Diaz-Decaro J Myers E Mucha J Neumann M Lewandowski W Kaczanowska M A systematic literature review of the economic and healthcare resource burden of cytomegalovirus. Curr Med Res Opin. (2023) 39(7):973–86. 10.1080/03007995.2023.2222583 37395088

[13] Tan YY Chan CY Sothirasan K Tan PL Maiwald M Thoon KC Outcomes of a targeted congenital cytomegalovirus infection screening approach among infants born ≥35 weeks gestation. Ann Acad Med Singapore. (2023) 52(11):643–4. 10.47102/annals-acadmedsg.2023254 38920153

[14] Wong A Tan KH Tee CS Yeo GS. Seroprevalence of cytomegalovirus, toxoplasma and parvovirus in pregnancy. Singapore Med J. (2000) 41(4):151–5. 11063178

[15] Lim SL Tan WC Tan LK. Awareness of and attitudes toward congenital cytomegalovirus infection among pregnant women in Singapore. Int J Gynecol Obstet. (2012) 117(3):268–72. 10.1016/j.ijgo.2011.12.025 22445422

[16] Kimberlin DW Jester PM Sánchez PJ Ahmed A Arav-Boger R Michaels MG Valganciclovir for symptomatic congenital cytomegalovirus disease. N Engl J Med. (2015) 372(10):933–43. 10.1056/NEJMoa1404599 25738669 PMC4401811

[17] Luck SE Wieringa JW Blázquez-Gamero D Henneke P Schuster K Butler K Congenital cytomegalovirus: a European expert consensus statement on diagnosis and management. Pediatr Infect Dis J. (2017) 36(12):1205–13. 10.1097/INF.0000000000001763 29140947

[18] Lanzieri TM Leung J Caviness AC Chung W Flores M Blum P Long-term outcomes of children with symptomatic congenital cytomegalovirus disease. J Perinatol. (2017) 37(7):875–80. 10.1038/jp.2017.41 28383538 PMC5562509

[19] Lim ERJ Ho SKY Chan DKL Yong TT Thong JF. Attitude towards screening for congenital cytomegalovirus infection innewborns in Singapore. Ann Acad Med Singapore. (2022) 51(6):373–5. 10.47102/annals-acadmedsg.2021459 35786758

[20] Ssentongo P Hehnly C Birungi P Roach MA Spady J Fronterre C Congenital cytomegalovirus infection burden and epidemiologic risk factors in countries with universal screening: a systematic review and meta-analysis. JAMA Netw Open. (2021) 4(8):e2120736. 10.1001/jamanetworkopen.2021.20736 34424308 PMC8383138

[21] Ross SA Novak Z Pati S Boppana SB. Overview of the diagnosis of cytomegalovirus infection. Infect Disord - Drug Targets. (2011) 11(5):466–74. 10.2174/187152611797636703 21827433 PMC3730495

[22] Alarcon A Martinez-Biarge M Cabañas F Quero J García-Alix A. A prognostic neonatal neuroimaging scale for symptomatic congenital cytomegalovirus infection. Neonatology. (2016) 110(4):277–85. 10.1159/000446690 27344149

[23] Gantt S Dionne F Kozak FK Goshen O Goldfarb DM Park AH Cost-effectiveness of universal and targeted newborn screening for congenital cytomegalovirus infection. JAMA Pediatr. (2016) 170(12):1173. 10.1001/jamapediatrics.2016.2016 27723885

[24] Leruez-Ville M Chatzakis C Lilleri D Blazquez-Gamero D Alarcon A Bourgon N Consensus recommendation for prenatal, neonatal and postnatal management of congenital cytomegalovirus infection from the European congenital infection initiative (ECCI). Lancet Reg Health - Eur. (2024) 40:100892. 10.1016/j.lanepe.2024.100892 38590940 PMC10999471

[25] Joint Committee on Infant Hearing. Year 2007 position statement: principles and guidelines for early hearing detection and intervention programs. Pediatrics. (2007) 120(4):898–921. 10.1542/peds.2007-2333 17908777

[26] Coppola T Mangold JF Cantrell S Permar SR. Impact of maternal immunity on congenital cytomegalovirus birth prevalence and infant outcomes: a systematic review. Vaccines (Basel). (2019) 7(4):129. 10.3390/vaccines7040129 31561584 PMC6963523

[27] Fowler KB. Congenital cytomegalovirus infection: audiologic outcome. Clin Infect Dis. (2013) 57(suppl_4):S182–4. 10.1093/cid/cit609 24257423 PMC3836573

[28] Yoshida H Takahashi H Kanda Y Kitaoka K Hara M. Long-term outcomes of cochlear implantation in children with congenital cytomegalovirus infection. Otol Neurotol. (2017) 38(7):e190–4. 10.1097/MAO.0000000000001483 28604578

[29] Lorenzoni F Lunardi S Liumbruno A Ferri G Madrigali V Fiorentini E Neonatal screening for congenital cytomegalovirus infection in preterm and small for gestational age infants. J Matern Fetal Neonatal Med. (2014) 27(15):1589–93. 10.3109/14767058.2013.871253 24328547

[30] De Vries JJC Van Der Eijk AA Wolthers KC Rusman LG Pas SD Molenkamp R Real-time PCR versus viral culture on urine as a gold standard in the diagnosis of congenital cytomegalovirus infection. J Clin Virol. (2012) 53(2):167–70. 10.1016/j.jcv.2011.11.006 22177273

[31] Lazzarotto T Blázquez-Gamero D Delforge ML Foulon I Luck S Modrow S Congenital cytomegalovirus infection: a narrative review of the issues in screening and management from a panel of European experts. Front Pediatr. (2020) 8:4. 10.3389/fped.2020.00013 32083040 PMC7006044

[32] Leruez-Ville M Magny JF Couderc S Pichon C Parodi M Bussières L Risk factors for congenital cytomegalovirus infection following primary and nonprimary maternal infection. Clin Infect Dis. (2017) 65(3):398–404. 10.1093/cid/cix337 28419213

[33] Kaye T Dufort EM Rosendahl SD Hullerman Umar J Pavan A Tricas K Universal newborn screening and surveillance for congenital cytomegalovirus — Minnesota, 2023–2024. MMWR Morb Mortal Wkly Rep. (2024) 73(32):703–5. 10.15585/mmwr.mm7332a2 39146269

[34] Wang L Xu X Zhang H Qian J Zhu J. Dried blood spots PCR assays to screen congenital cytomegalovirus infection: a meta-analysis. Virol J. (2015) 12(1):60. 10.1186/s12985-015-0281-9 25889596 PMC4408583

[35] Inoue N Koyano S. Evaluation of screening tests for congenital cytomegalovirus infection. Pediatr Infect Dis J. (2008) 27(2):182–4. 10.1097/INF.0b013e318161a2d5 18174856

[36] Chiereghin A Pavia C Turello G Borgatti EC Baiesi Pillastrini F Gabrielli L Universal newborn screening for congenital cytomegalovirus infection – from infant to maternal infection: a prospective multicenter study. Front Pediatr. (2022) 10:909646. 10.3389/fped.2022.909646 35874574 PMC9298552

[37] Wilson J Jungner G, World Health Organization. Principles and practice of screening for disease. Public Health Pap World Health Organ. (1968) 2:21. https://iris.who.int/handle/10665/208882

[38] Dobrow MJ Hagens V Chafe R Sullivan T Rabeneck L. Consolidated principles for screening based on a systematic review and consensus process. CMAJ. (2018) 190(14):E422–9. 10.1503/cmaj.171154 29632037 PMC5893317

[39] Pata D Buonsenso D Turriziani-Colonna A Salerno G Scarlato L Colussi L Role of valganciclovir in children with congenital CMV infection: a review of the literature. Children. (2023) 10(7):1246. 10.3390/children10071246 37508743 PMC10378502

[40] Chung PK Schornagel F Oudesluys-Murphy AM De Vries LS Soede W Van Zwet E Targeted screening for congenital cytomegalovirus infection: clinical, audiological and neuroimaging findings. Arch Dis Child - Fetal Neonatal Ed. (2023) 108(3):302–8. 10.1136/archdischild-2022-324699 36549893 PMC10176415

[41] Diener ML Zick CD McVicar SB Boettger J Park AH. Outcomes from a hearing-targeted cytomegalovirus screening program. Pediatrics. (2017) 139(2):e20160789. 10.1542/peds.2016-0789 28119425

[42] Williams EJ Kadambari S Berrington JE Luck S Atkinson C Walter S Feasibility and acceptability of targeted screening for congenital CMV-related hearing loss. Arch Dis Child - Fetal Neonatal Ed. (2014) 99(3):F230. 10.1136/archdischild-2013-305276 24596404

[43] Fowler KB McCollister FP Sabo DL Shoup AG Owen KE Woodruff JL A targeted approach for congenital cytomegalovirus screening within newborn hearing screening. Pediatrics. (2017) 139(2):e20162128. 10.1542/peds.2016-2128 28049114 PMC5260148

[44] Schleiss MR. Evolving concepts in care for congenital cytomegalovirus (cCMV) infection: better outcomes for more babies!. J Pediatr. (2024) 270:114044. 10.1016/j.jpeds.2024.114044 38575056

[45] Schleiss MR. Congenital cytomegalovirus: impact on child health. Contemp Pediatr. (2018) 35(7):16–24. 30740598 PMC6368255

[46] Gillespie AN Dalziel K Webb E Wong J Jones CA Sung V Targeted screening for congenital cytomegalovirus: a micro-costing analysis. J Paediatr Child Health. (2023) 59(1):64–71. 10.1111/jpc.16239 36250562 PMC10946837

[47] Grosse SD Dollard SC Ortega-Sanchez IR. Economic assessments of the burden of congenital cytomegalovirus infection and the cost-effectiveness of prevention strategies. Semin Perinatol. (2021) 45(3):151393. 10.1016/j.semperi.2021.151393 33551180 PMC8335728

[48] Retzler J Hex N Bartlett C Webb A Wood S Star C Economic cost of congenital CMV in the UK. Arch Dis Child. (2019) 104(6):559. 10.1136/archdischild-2018-316010 30472664

[49] Ronchi A Shimamura M Malhotra PS Sánchez PJ. Encouraging postnatal cytomegalovirus (CMV) screening: the time is NOW for universal screening!. Expert Rev Anti Infect Ther. (2017) 15(5):417–9. 10.1080/14787210.2017.1303377 28277819

[50] Din ES Brown CJ Grosse SD Wang C Bialek SR Ross DS Attitudes toward newborn screening for cytomegalovirus infection. Pediatrics. (2011) 128(6):e1434–42. 10.1542/peds.2011-1444 22084323

[51] Phillips VL Xu J Park A Gantt S Dedhia K. The cost-effectiveness of targeted screening for congenital cytomegalovirus in newborns compared to clinical diagnosis in the US. Int J Pediatr Otorhinolaryngol. (2023) 166:111450. 10.1016/j.ijporl.2023.111450 36773448

